# Expression of Biofilm-Degrading Enzymes in Plants and Automated High-Throughput Activity Screening Using Experimental *Bacillus subtilis* Biofilms

**DOI:** 10.3389/fbioe.2021.708150

**Published:** 2021-09-21

**Authors:** P. Opdensteinen, S. J. Dietz, B. B. Gengenbach, J. F. Buyel

**Affiliations:** ^1^ Fraunhofer Institute for Molecular Biology and Applied Ecology IME, Aachen, Germany; ^2^ Institute for Molecular Biotechnology, RWTH Aachen University, Aachen, Germany

**Keywords:** assay development, microbial growth inhibition, pathogenic bacteria, plant cell packs, plant molecular farming

## Abstract

Biofilm-forming bacteria are sources of infections because they are often resistant to antibiotics and chemical removal. Recombinant biofilm-degrading enzymes have the potential to remove biofilms gently, but they can be toxic toward microbial hosts and are therefore difficult to produce in bacteria. Here, we investigated *Nicotiana* species for the production of such enzymes using the dispersin B-like enzyme *Lysobacter gummosus* glyco 2 (Lg2) as a model. We first optimized transient Lg2 expression in plant cell packs using different subcellular targeting methods. We found that expression levels were transferable to differentiated plants, facilitating the scale-up of production. Our process yielded 20 mg kg^−1^ Lg2 in extracts but 0.3 mg kg^−1^ after purification, limited by losses during depth filtration. Next, we established an experimental biofilm assay to screen enzymes for degrading activity using different *Bacillus subtilis* strains. We then tested complex and chemically defined growth media for reproducible biofilm formation before converting the assay to an automated high-throughput screening format. Finally, we quantified the biofilm-degrading activity of Lg2 in comparison with commercial enzymes against our experimental biofilms, indicating that crude extracts can be screened directly. This ability will allow us to combine high-throughput expression in plant cell packs with automated activity screening.

## Introduction

Many bacteria, including pathogens (Dufour et al., 2010), naturally form biofilms (Stiefel et al., 2016) to protect themselves against environmental threats such as UV light, disinfectants, antibiotics, and host immune effector mechanisms (Del Pozo, 2018). Harsh mechanical, physical or chemical treatments are often required to remove biofilms, but they are unsuitable for sensitive medical instruments such as endoscopes, leaving them susceptible to bacterial colonialization (Stiefel et al., 2016). Harsh methods are also unsuitable for the removal of biofilms formed by pathogens inside the human body, contributing to chronic wounds, persistent infections, and malfunctioning medical devices (Metcalf and Bowler, 2013; Del Pozo, 2018).

As an alternative to chemical and mechanical methods, enzymes can facilitate the dispersion of biofilms under mild conditions, including physiological temperatures (Stiefel et al., 2016). Such enzymes target the major components of biofilms, namely, exopolysaccharides (EPS), proteins, and nucleic acids (Roux et al., 2015). For example, broad-spectrum proteases such as proteinase K can be used to degrade protein components, whereas DNase I can break down the extracellular DNA (Izano et al., 2008) that contributes to biofilm stability (Kaplan, 2009). However, the efficiency of these enzymes varies depending on the composition of the biofilm, which in turn depends on the microorganisms, growth conditions, and environment (Roux et al., 2015).

The main component of many bacterial biofilms is poly-*β*-1,6-*N*-acetyl-d-glucosamine (PNAG) (Jabbouri and Sadovskaya, 2010). This EPS is highly conserved among diverse bacteria, including pathogens such as *Staphylococcus aureus* (Kaplan, 2009; Cywes-Bentley et al., 2013). It thus makes a promising target for the dispersion of microbial biofilms, but only two enzymes that can hydrolyze this substrate have been described thus far: dispersin B (DspB), which was discovered in *Aggregatibacter actinomycetemcomitans* (Kaplan et al., 2003), and PgaB, which was discovered in *Bordetella bronchiseptica* (Little et al., 2018). Facultative predatory microbes that lyse other bacteria may provide a source of novel biofilm-degrading enzymes, including species from the genera *Myxobacteria* (Muñoz-Dorado et al., 2016) and *Lysobacter* (Gökçen et al., 2014). The *Lysobacter gummosus* genome was recently sequenced, aiming to identify candidate enzymes with biofilm-degrading activity (Gökçen et al., 2014).

The production of recombinant biofilm-degrading enzymes has been attempted in microbes such as *Escherichia coli* (Dobrynina et al., 2015; Cherdvorapong et al., 2020), but this is challenging because the inherent antibacterial properties of these products can interfere with host cell growth (Oey et al., 2009). Plants such as *Nicotiana benthamiana* and tobacco (*Nicotiana tabacum*) are promising alternative hosts that benefit from inexpensive and rapidly scalable upstream production as well as product yields of more than 4 g kg^−1^ fresh leaf biomass (Yamamoto et al., 2018) combined with high biomass yields of ∼100,000 kg ha^−1^ y^−1^ (*N. tabacum*) (Stoger et al., 2002; Buyel et al., 2017)]. Plants are also unable to support the replication of human viruses, therefore increasing the safety profile of recombinant proteins administered to humans (Richard et al., 2013). Importantly, minimal processing is sufficient if plant-derived enzymes are used for the cleaning of instruments and other surfaces outside the human body (Rosenberg et al., 2015), which reduces the costs of downstream processing. Although biofilm-degrading enzymes have already been expressed in transgenic plants to protect them from pathogens (Ragunath et al., 2012), the transient expression of such enzymes for technical or pharmaceutical applications has not been studied in detail.

Here, we optimized the upstream production of the dispersin B–like enzyme *L. gummosus* glyco 2 (Lg2) in plant cell packs (PCPs) (Gengenbach et al., 2020) and transferred the optimal conditions to differentiated *N. benthamiana* plants for transient expression. We then developed an immobilized metal affinity chromatography (IMAC) protocol for the purification of Lg2. As proof of principle, we developed a convenient and automated assay to screen candidate enzymes for biofilm-degrading activity and investigated the activity of Lg2 against experimental *Bacillus subtilis* biofilms in the assay, to establish a high-throughput approach for the screening of novel biofilm-degrading enzymes. The IMAC protocol exploited the presence of a His_6_ tag, which has a small size and charge and thus has little impact on the catalytic activity of recombinant proteins when placed on the C-terminus or the N-terminus. This should allow our purification protocol to be used with other biofilm-degrading enzymes in the future (Terpe, 2003).

## Materials and Methods

### Cloning of Expression Constructs

The coding sequence of the dispersin B homolog Lg2 from *L. gummosus* (Gökçen, 2016) was modified to incorporate flanking N-terminal BspHI and C-terminal NotI sites before codon optimization for *N. benthamiana* and synthesis by GeneArt (Thermo Fisher Scientific, Darmstadt, Germany). Using the BspHI and NotI sites, the Lg2 gene was subcloned into 12 previously established pTRA vectors (Gengenbach et al., 2020), originally derived from pPAM (GenBank AY027531), featuring all possible permutations (Supplementary Table S1) of the CHS, omega, and TL 5’ untranslated regions (UTRs) combined with an LPH signal sequence targeting the secretory pathway, an *rbcs* signal sequence targeting the chloroplast, a SEKDEL ER retention signal, or none of the above to allow accumulation in the cytosol (Buyel et al., 2013). Plasmids were propagated in *E. coli* DH5α and transferred to *Agrobacterium tumefaciens* (*Rhizobium radiobacter*) GV3101:pMP90RK by electroporation (2400 V, 25 μF, and 200 Ω) using 0.2 cm electroporation cuvettes (Bio-Rad Laboratories, Hercules, California, United States) as previously described (Main et al., 1995).

### *Agrobacterium* Cultivation and Infiltration of Plant Cell Packs and Differentiated Plants

For transient expression, *A. tumefaciens* precultures were inoculated from glycerol stocks to an OD_600nm_ of 0.04 in PAM4 medium (Houdelet et al., 2017) containing 50 mg L^–1^ carbenicillin, 25 mg L^−1^ kanamycin, and 25 mg L^−1^ rifampicin and cultivated for 24 h at 28°C and 160 rpm (500 mL in a baffled Fernbach flask for the infiltration of differentiated plants) or 1,000 rpm (500 µL well^−1^ in 96 deepwell plates for the infiltration of PCPs). Main cultures were inoculated from the precultures to an OD_600nm_ of 0.1 and were incubated for 24 h using the same medium and cultivation conditions.

The automated infiltration of PCPs with *A. tumefaciens* was carried out as previously described (Gengenbach et al., 2020) using 100 µL infiltration solution per PCP [0.5 g L^−1^ Murashige and Skoog (MS) major and minor salts mixture, 50.0 g L^−1^ (146 mM) sucrose, 2.0 g L^−1^ (10 mM) glucose monohydrate, 0.0392 g L^−1^ (0.2 mM) acetosyringone, and 2.928 g L^−1^ (15 mM) 2-(*N*-morpholino)ethanesulfonic acid (MES); pH 5.6] with an OD_600nm_ of 0.4. Infiltrated PCPs were incubated for 72 h at 26°C and 80% relative humidity in an inverted position over a water reservoir (Gengenbach et al., 2020).

For the infiltration of differentiated plants, infiltration solutions were prepared by diluting *Agrobacterium* cultures with water and infiltration buffer to an OD_600nm_ of 0.5 [final concentration of 0.5 g L^−1^ Fertilizer MEGA 2 and 0.0392 g L^−1^ (0.2 mM) acetosyringone; pH 5.6]. Whole plants were infiltrated by submerging the stem and leaves in the infiltration suspension, applying a vacuum (100 mbar) for 1 min, and rapidly releasing the vacuum. Infiltrated plants were inverted and incubated for 5 days as previously described (Menzel et al., 2016). Before the infiltration of PCPs or differentiated plants, the *Agrobacterium* infiltration solutions were induced for 1 h.

### Cultivation and Extraction of Plant Cell Packs and Differentiated Plants

Seven-week-old *N. benthamiana* and *N. tabacum* plants, cultivated on stonewool blocks in a phytotron as previously described (Menzel, 2018), were used for all transient expression studies in differentiated plants. Whole plants were extracted using 3 v m^−1^ ratio of extraction buffer (50 mM sodium phosphate buffer, 10 mM sodium bisulfite, and 500 mM sodium chloride; pH 8.0) in a blender (Koninklijke Philips, Amsterdam, Netherlands) for 3 × 30 s with 30 s breaks between mixing cycles as previously described (Buyel et al., 2014). Samples were centrifuged twice at 16,000 × g, for 20 min at 4°C, and the supernatant was stored at −20°C.

*N. tabacum* BY-2 cells [100 g wet biomass L^−1^ and packed cell volume of 30–40% (v v^−1^)] were cultivated in continuous 5 L suspension cultures (Holland et al., 2010) and were concentrated twofold for the preparation of PCPs by sedimentation as previously described (Gengenbach et al., 2020). PCPs (60 mg PCP^−1^) were extracted using 3 v m^−1^ ratio of the same extraction buffer in 1.2 mL collection microtube strips (Qiagen, Hilden, Germany) containing a single 3 mm steel bead per well in a MM 300-bead mill (Retsch, Han, Germany) at 28 Hz for 2 × 3 min (Gengenbach et al., 2020). Extracts were clarified by centrifugation at 5,100 × g for 8 min at 4°C, and supernatants were stored at −20°C.

### Cultivation, Degradation, and Staining of Experimental *B. subtilis* Biofilms

The experimental biofilms were based on wild-type *B. subtilis* or the knockout strain WB800N lacking eight extracellular proteinases (Jeong et al., 2018). *B. subtilis* precultures [50 mL terrific broth (TB; 11.8  L^−1^ tryptone, 23.6 g L^−1^ yeast extract, 9.4 g L^−1^ dipotassium hydrogen phosphate, 2.2 g L^−1^ potassium dihydrogen phosphate, and 4.5 g L^−1^ glycerol) in 500 mL nonbaffled glass flasks] were cultivated at 37°C and 160 rpm to an OD_600nm_ of ∼5.0 and harvested by centrifugation at 3,200 × g for 5 min at ∼22°C. The cell pellet was resuspended in 25 mL MSgg medium [5 mM potassium phosphate, 100 mM 3-(*N*-morpholino)propanesulfonic acid (MOPS), 2 mM magnesium chloride, 0.7 mM calcium chloride, 50 µM manganese chloride, 50 µM ferric chloride (FeCl_3_), 1 µM zinc chloride, 2 µM thiamine, 5.6 g L^−1^ glycerol, 5.0 g L^−1^ glutamate, 50 μg mL^−1^ tryptophan, 50 μg mL^−1^ phenylalanine, and 50 μg mL^−1^ threonine; pH 7.0 (Branda et al., 2001)] and diluted to an OD_600nm_ of 0.1 with MSgg, and 200 µL of the cell suspension was dispensed into each well of a Cellstar flat-bottom 96-well plate (lot: 07460135, Greiner BioOne, Kremsmünster, Austria). Blanks were filled with 200 µL sterile MSgg medium. Plates were sealed with gas-permeable membranes with vapor transmission rates of 450 g m^−2^ d^−1^ (4titude, Wotton, United Kingdom) or 700 g m^−2^ d^−1^ (Diversified Biotech/Sigma-Aldrich, St Louis, MO, United States), to control evaporation, and were incubated for 24 h at 37°C without agitation. The supernatant was discarded, and the preformed biofilms were incubated with proteinase K (Sigma-Aldrich), DNase I (Sigma-Aldrich), or Lg2 in 200 µL assay buffer as required. For proteinase K treatment, we used an assay buffer comprising 50 mM tris(hydroxymethyl)aminomethane (TRIS) for pH 7.0–9.0 or 50 mM MES for pH 5.0–6.5, in each case combined with 5 mM calcium chloride. The same assay buffers supplemented with 5 mM magnesium chloride were used for DNase I. For biofilm degradation with Lg2, we used a phosphate-based buffer solution [50 mM sodium phosphate and 100 mM sodium chloride (Chaignon et al., 2007)]. Plates were not agitated during the enzymatic treatment.

Following the enzymatic treatment, the supernatants were discarded and the biofilm was washed twice with 220 µL deionized water per well. Each well was then filled with 200 µL 0.1% m v^−1^ crystal violet (Carl Roth, Karlsruhe, Germany) and incubated for 5 min at 22°C to stain the remaining biofilm (Stepanović et al., 2000), followed by washing twice with 220 µL deionized water per well. The plates were air dried for 10 min before adding 30% v v^−1^ acetic acid (Stepanović et al., 2000), thorough mixing, and incubation for 15 min at 22°C on a rotary shaker at 300 rpm. Signals were detected at 595 nm using an Enspire plate reader (PerkinElmer, Waltham, MA, United States).

### Statistical Design of Experiments

Design‐Expert v13 (Stat‐Ease, Minneapolis, MN, United States) was used to set up and analyze all models described herein. The effect of partially purified Lg2 (IMAC eluate) on experimental *B. subtilis* (wild-type) biofilms was investigated using an I-optimal response surface deign with 24 runs. The DoE covered pH values in the range of 5.0–9.0, Lg2 concentrations in the range of 10–70 μg mL^−1^, and incubation periods in the range of 2–8 h. Control experiments with the reference biofilm-degrading enzymes proteinase K and bovine DNase I were conducted with the same strain in a D-optimal combined mixture design with 153 runs conducted in three blocks. The DoE covered pH values in the range of 5.0–9.0, proteinase K and bovine DNase I concentrations in the range of 10–500 μg mL^−1^, and incubation periods in the range of 2–24 h. An additional I‐optimal response surface split-plot design with membrane permeability as a hard-to-change factor with 128 runs was conducted in two blocks using experimental biofilms formed by the *B. subtilis* mutant strain WB800N and covering similar pH ranges, enzyme concentrations, and incubation periods as above. We used two membranes featuring evaporation rates of 544 or 915 g m^−2^ d^−1^, and the evaporation of water over time was quantified from preweighted plates filled with deionized water and sealed with these membranes.

### Automation

A JANUS G3 liquid-handling station (PerkinElmer, Waltham, MA, United States) was used to automate the biofilm-degradation assay. Experimental designs generated with Design‐Expert v13 were converted to *.csv (MS-DOS) worklists for 1) pipetting enzyme solutions and 2) removing them followed by two washing steps (200 µL assay buffer without enzyme) for all time points required by the DoE. Herein, the run numbers generated by the DoE were converted to well positions in a 96-well plate to ensure a randomized order of experiments within the plates. Next, biofilms prepared in Greiner BioOne 96-well plates as well as stock solutions for all enzymes were placed at predefined positions on the deck of the liquid-handling station. Enzyme solutions (200 µL well^−1^) were automatically added to the 96-well plate containing the preformed biofilms as specified in the worklists before sealing the plate with a 96-well Robolid Corner Notch lid (Corning, Corning, NY, United States). The sealed plate was then transferred to a thermoshake heated orbital shaker (Inheco, Planegg, Germany) to maintain a constant incubation temperature of 37°C without agitations. Following incubation for the time points specified in the worklists, wells were washed twice with 200 µL assay buffer and filled with 200 µL assay buffer (without enzyme) to avoid dehydration of the biofilms before staining. Biofilms incubated with 200 µL assay buffer lacking the enzyme were washed at the same time points in a similar manner to generate authentic controls. Finally, the treated biofilms were stained and quantified as described above for the manual assay.

### Sample Analysis

The concentration of total soluble protein in samples was determined using the Bradford method (Simonian and Smith, 2006) as previously described (Buyel and Fischer, 2014). In brief, 195 µL Bradford reagent (Thermo Fisher Scientific) was mixed with 5 µL sample or the bovine serum albumin standard in the range 0–2000 mg L^−1^ in phosphate-buffered saline (PBS). After incubating for 10 min at 22°C, the absorbance of the dye–protein complex was measured at 595 nm using an Enspire plate reader. Expression levels were estimated initially by LDS-PAGE as previously described (Menzel et al., 2016). Coomassie-stained LDS-PAGE gels were analyzed using an AIDA Image Analyzer (Raytest, Straubenhardt, Germany), and the peak area for each band was used to estimate the abundance of Lg2 or host cell proteins during purification.

The concentration of His_6_-tagged Lg2 was quantified by dot blot analysis. In brief, 5 µL of clarified PCP or plant extract as well as eight standards of purified Lg2 in the range 0.5–15.0 mg L^−1^ in PBS were pipetted onto an Amersham Protran nitrocellulose membrane (Sigma‐Aldrich). Membranes were air-dried for 5 min and blocked with 5% m v^−1^ milk powder (Roth, Karlsruhe, Germany) in PBS supplemented with 0.05% v v^−1^ Tween-20 (PBST) for 1 h on a rotary shaker at 22°C. The membranes were incubated with a polyclonal rabbit anti-His antibody (GenScript Biotech, Piscataway, NJ, United States) at a concentration of 0.1 mg L^−1^ in 5% m v^−1^ milk power in PBST and with alkaline phosphatase (AP)-labeled goat anti-rabbit antibody (Jackson ImmunoResearch, West Grove, PA, United States) at a concentration of 0.06 mg L^−1^ in 5% m v^−1^ milk powder for 1 h each. Finally, the AP signal was detected by adding NBT/BCIP (Carl Roth) as previously described (Kastilan et al., 2017). Membranes were washed twice for 5 min each with PBST between incubation steps. The AP signal was quantified by densitometric analysis using ImageJ (Schneider et al., 2012) software (National Institutes of Health, Bethesda, MD, United States).

## Results and Discussion

### High-Throughput Expression Screening of Lg2 in Plant Cell Packs and Scale-Up of Production in Differentiated Plants

**FIGURE 1:**
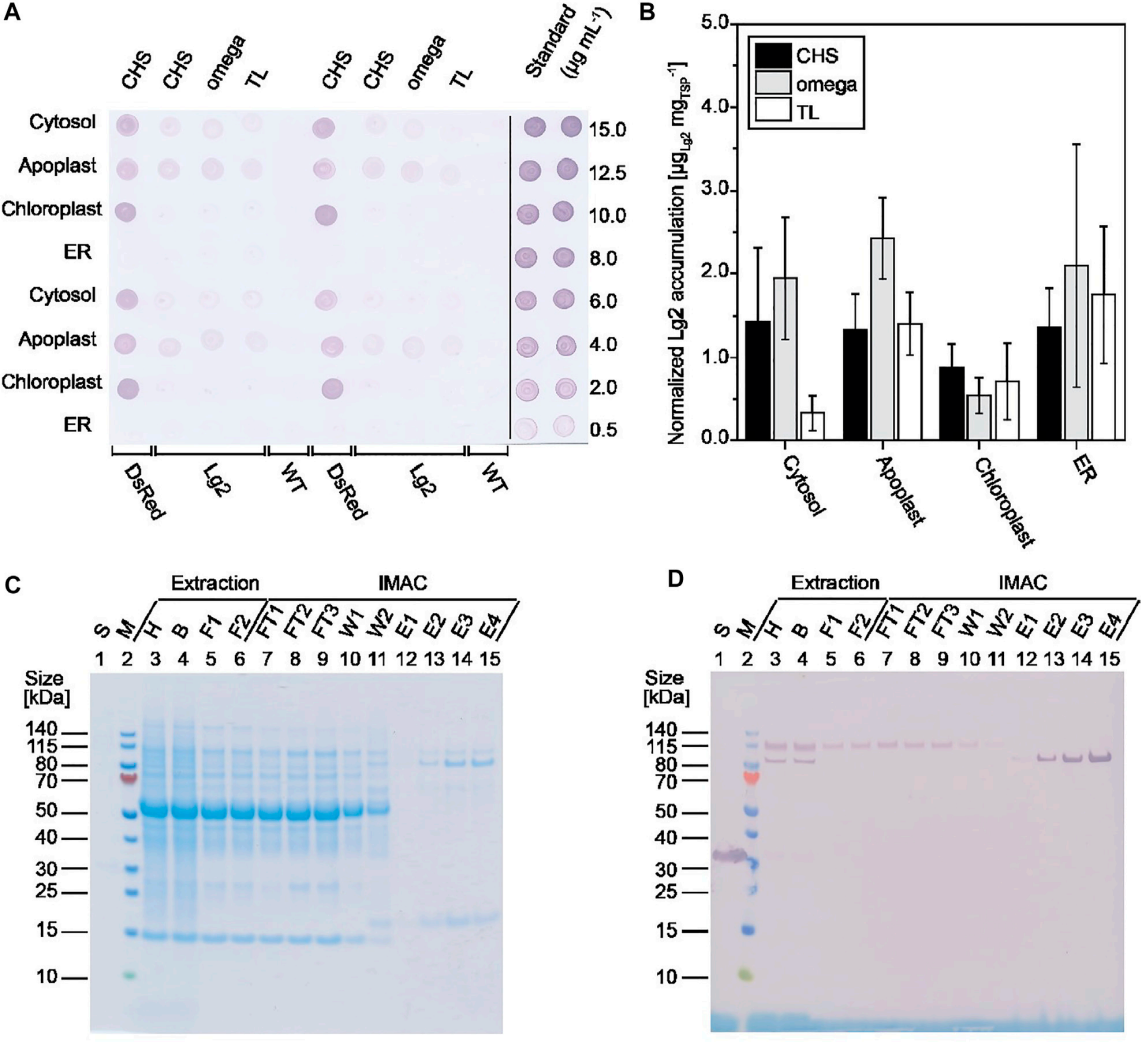
Screening of different Lg2 constructs for expression. **(A)** Lg2 accumulation in BY-2 PCPs at three dpi as detected by dot blot using a His6-specific primary antibody and an AP-labeled goat anti-rabbit secondary antibody. **(B)** Lg2 accumulation in *N. benthamiana* plants at five dpi based on densiometric evaluation of dot blots using a His6-specific and AP-labeled antibody, normalized to total soluble protein. Data are means ± standard deviation for n = 3 (or n = 6 for ER constructs) individual plants. **(C, D)** Analysis of samples from Lg2 purification after extraction in 50 mM disodium phosphate, 10 mM sodium bisulfite, and 500 mM sodium chloride (pH 8.0). Samples were analyzed by LDS-PAGE followed by staining with Coomassie Brilliant Blue **(C)** or western blot using a His6-specific and AP-labeled antibody **(D)**. The observed size of purified Lg2 on the gel and blot (∼85 kDa) matched the predicted size of 86.7 kDa. B, bag filtrate; E, IMAC eluate; F1, PDH4 depth-filter filtrate; F2, 0.2 µm sterile filtrate; FT, IMAC flowthrough; H, homogenate; S, standard (10 μg mL^−1^ His-tagged DsRed); W1, IMAC wash 1 (20 mM sodium phosphate, 500 mM sodium chloride; pH 7.5); W2, IMAC wash 2 (20 mM sodium phosphate, 500 mM sodium chloride, 15 mM imidazole; pH 7.5); WT, wild-type PCP extract.

We used our previously described automated high-throughput platform based on transient expression in PCPs prepared from tobacco BY-2 cells (Gengenbach et al., 2020) to systematically investigate the expression of C-terminally His_6_-tagged Lg2 in the cytosol, apoplast, ER, and chloroplasts, in each case using three different 5’ UTRs (Supplementary Table S1). Lg2 accumulated to the highest levels in the cytosol and apoplast (3.2 ± 0.8 mg kg^−1^, n = 4; Supplementary Figure S1), in the latter case when combined with the CHS 5’ UTR (Figure 1A). This is consistent with previous studies reporting the benefits of the CHS 5’ UTR for product accumulation (Buyel et al., 2013). As neither authentic nor homology structures of Lg2 are available, we were unable to determine the specific factors affecting its accumulation in different compartments, such as the pI based on surface-exposed amino acids or the accessibility of protease cleavage sites (Hehle et al., 2011; Martinière et al., 2013).

Having established that Lg2 accumulates in PCPs, we tested its expression in differentiated *N. benthamiana* plants (Figure 1B). We found that Lg2 levels in the cytosol, apoplast, and chloroplast matched the pattern observed in PCPs (Figure 1A). In contrast, Lg2 accumulated in the ER to substantially higher levels (up to 20 mg kg^−1^) in plants than in PCPs, perhaps because BY-2 cells are derived from *N. tabacum* roots rather than *N. benthamiana* leaves. PCPs generated from the latter could be developed in the future to avoid this discrepancy. Again, it is unclear whether the near-neutral pH in the cytosol, ER, and apoplast led to the generally higher accumulation of Lg2 in these compartments than in the more acidic chloroplasts (Elghabi et al., 2011; Martinière et al., 2013; Martinière et al., 2018). This highlights the importance of testing different subcellular targeting strategies during transient expression, which is not yet investigated routinely (Kovalskaya et al., 2016; Kovalskaya et al., 2019). The coexpression of a silencing suppressor such as p19 could also be used to increase product accumulation (Garabagi et al., 2012).

Having identified the apoplast as the most suitable compartment for the transient expression of Lg2, we captured the product from clarified extracts by IMAC (Figures 1C, D). We found that washing with a buffer solution containing 15 mM imidazole was sufficient to remove 69% of the host cell proteins that bound to the nickel-charged IMAC resin, although a major impurity of ∼17 kDa remained (Figure 1C, lanes 13–15). The purity of Lg2 in the eluate was 46% based on densitometric evaluation. However, the yield of Lg2 was 0.30 mg kg^−1^ wet plant biomass, equivalent to an overall recovery of only ∼1%, so further process development is necessary. We found that ∼90% product loss occurred during depth filtration potentially due to the presence of protein-binding diatomaceous earth in the filter material, but this could be reduced to <50% by using a glass fiber filter instead (our preliminary data). Importantly, we noted that depth filtration selectively removed ∼85 kDa Lg2 product that bound to the IMAC resin, but an Lg2 isoform of ∼115 kDa that did not bind to the resin was able to pass through the filter, also contributing to the overall low recovery (Figures 1C,D). The major impurity with a molecular mass of ∼17 kDa was successfully removed by size-exclusion chromatography (SEC) using a Sephacryl S100 resin, but this should be replaced by a membrane filtration step in the future to improve scalability (Opdensteinen et al., 2019).

### A High-Throughput Assay for the Screening of Candidate Biofilm-Degrading Enzymes

**FIGURE 2:**
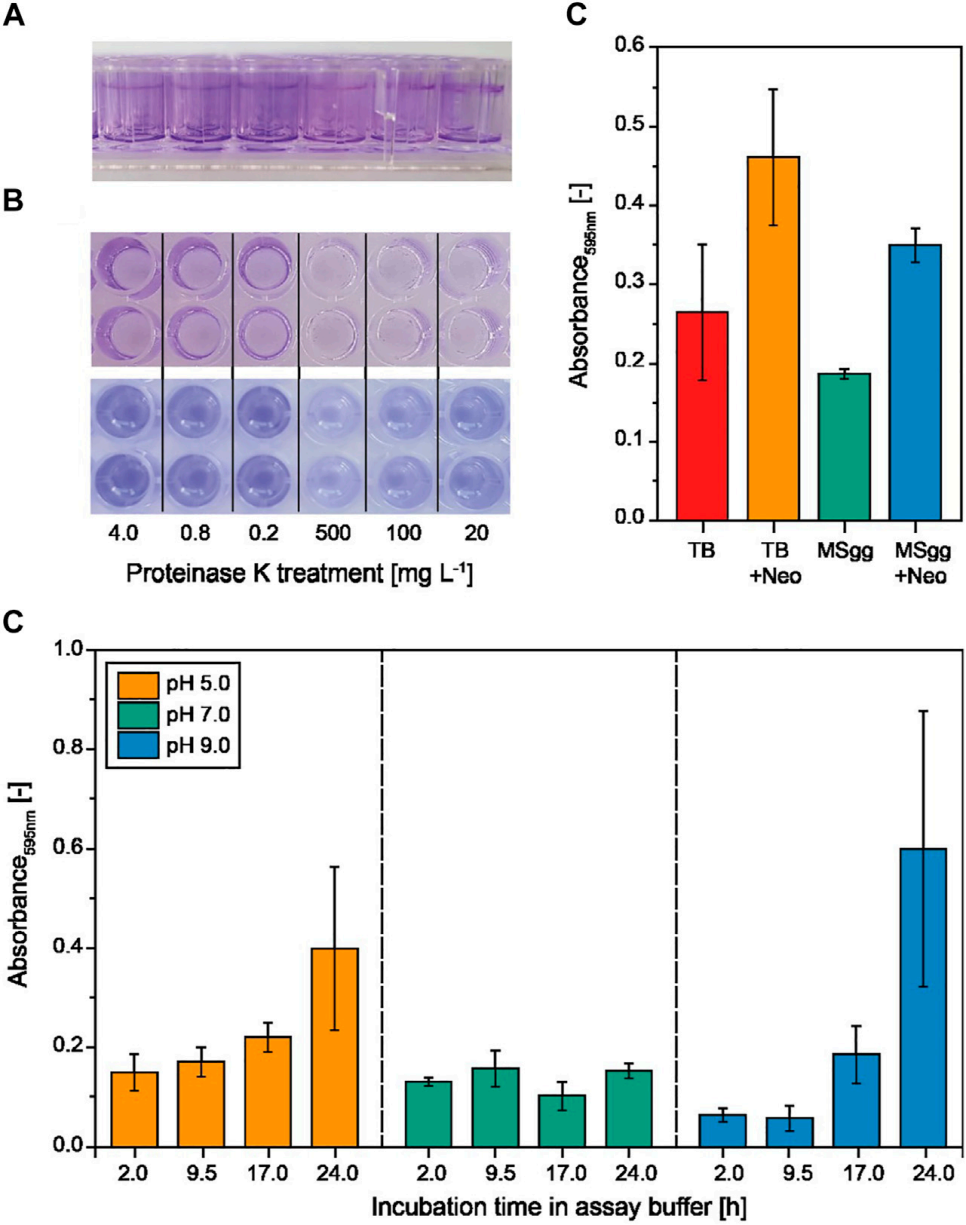
Formation and quantification of experimental *B. subtilis* biofilms in multiwell plates using different media and assay buffers. **(A)** Biofilms that were submerged (hydrophilic) and formed at the air–liquid interface (hydrophobic) in MSgg (Branda et al., 2001) and TB (not shown) after 24 h were stained with crystal violet. **(B)** Proteinase K–treated biofilms after staining (top) and after re-solubilization (bottom) of crystal violet with acetic acid for quantification in a plate reader. **(C)** The crystal violet staining intensity of the biofilms formed in MSgg or TB in the presence or absence of neomycin (Neo) measured at 595 nm. Data are means ± standard deviations (n = 6 biological replicates). **(D)** Incubation of preformed (24 h in MSgg) *B. subtilis* biofilms in degradation assay buffer (50 mM TRIS or MES, 5 mM calcium chloride) at different pH values and for different incubation periods (n = 3).

We used *B. subtilis* as a model because it forms submerged (hydrophilic) biofilms on the well base and walls as well as hydrophobic biofilms at the air–liquid interface (Figures 2A,B). These structures also contain the characteristic polymers of microbial biofilms, namely, (extracellular) DNA (Peng et al., 2020), proteins (Vlamakis et al., 2013), and EPS (Roux et al., 2015), thus allowing for different classes of biofilm-degrading enzymes to be tested in the same assay. For example, protein components such BslA are targeted by proteinases (Kobayashi and Iwano, 2012), whereas EPSs such as PNAG are targeted by carbohydrate-hydrolyzing enzymes. Moreover, *B. subtilis* can tolerate a broad pH range [<5 to >9 (Wilks et al., 2009)], allowing for biofilm-degrading enzymes to be tested across multiple environmental conditions.

We first tested complex TB and the chemically defined MSgg medium (Branda et al., 2001), each in the presence or absence of neomycin (Jeong et al., 2018), to identify conditions for the reproducible formation of *B. subtilis* biofilms before transferring the system to a high-throughput format. We observed a significantly higher variance (*p* < 0.01, *F*-test, *α* = 0.05, n = 6) for the amount of biofilms (measured as absorbance following crystal violet staining) formed in the complex TB medium than in the MSgg medium (Figure 2C). Interestingly, the addition of neomycin increased the absorbance signal by >40%, probably reflecting a stress response, and in the case of MSgg, neomycin simultaneously increased the standard deviation by >50%. Our focus was reproducibility during the formation of experimental biofilms, so we selected MSgg without antibiotics because it was associated with the lowest variability. We then incubated the biofilms with assay buffer from the biofilm-degrading enzymes (Figure 2D, Supplementary Figure S2). We observed significantly greater signal variability (1) at the pH extremes (5.0 and 9.0) after 24 h of incubation compared to a neutral assay buffer and (2) after incubation for 24 h at pH 9.0 compared to shorter incubation time periods (Supplementary Table S2). This was not related to an increase in osmolality due to evaporation over time, because a similar effect was observed when we supplemented the assay buffer with 100 mM sodium chloride (Supplementary Figure S2). The assay buffer with the lowest impact on the experimental biofilm appeared to be a phosphate buffer as indicated by the slope of linear regression curves fitted for every pH value and incubation time tested (Supplementary Table S3). The buffering range of phosphate is not ideal at pH 5.0 or 9.0; however, only minimal pH changes during biofilm treatment were expected due to the lack of a carbon source for bacterial growth. In order to compensate for the influence of the assay buffer on the experimental biofilms, we included controls for all pH values and incubation periods in subsequent experiments.

### Degradation of Experimental Biofilms Using Plant-Derived Lg2 and Combinations of Different Enzyme Classes

Having established reproducible conditions for biofilm formation, we tested the activity of both crude Lg2-containing extracts and IMAC–purified Lg2 on *B. subtilis* biofilms. The quantity of biofilms remaining in the wells was significantly reduced by ∼75% following treatment with extracts containing Lg2 compared to wild-type extracts as controls (*p* = 0.023, two-sided two-sample *t*-test, *α* = 0.05, n = 4; Supplementary Figure S3). The plant-derived enzyme was therefore considered active, as previously reported for Lg2 expressed in *E. coli* (Gökçen, 2016). There was also a statistically significant difference (*p* < 0.001, two-sided two-sample *t*-test, *α* = 0.05, n = 4) between untreated biofilms and those treated with mock PCP extracts, which was attributed to the different assay buffers used (Supplementary Figure S3) (Ragunath et al., 2012). This suggested that PCP extracts containing recombinant candidate enzymes can be used directly to screen for biofilm-degrading activity, as previously shown for extracts from differentiated plants (Ragunath et al., 2012). The direct application of extracts can simplify sample preparation and increase screening throughput by facilitating automation (Gengenbach et al., 2020).

**FIGURE 3:**
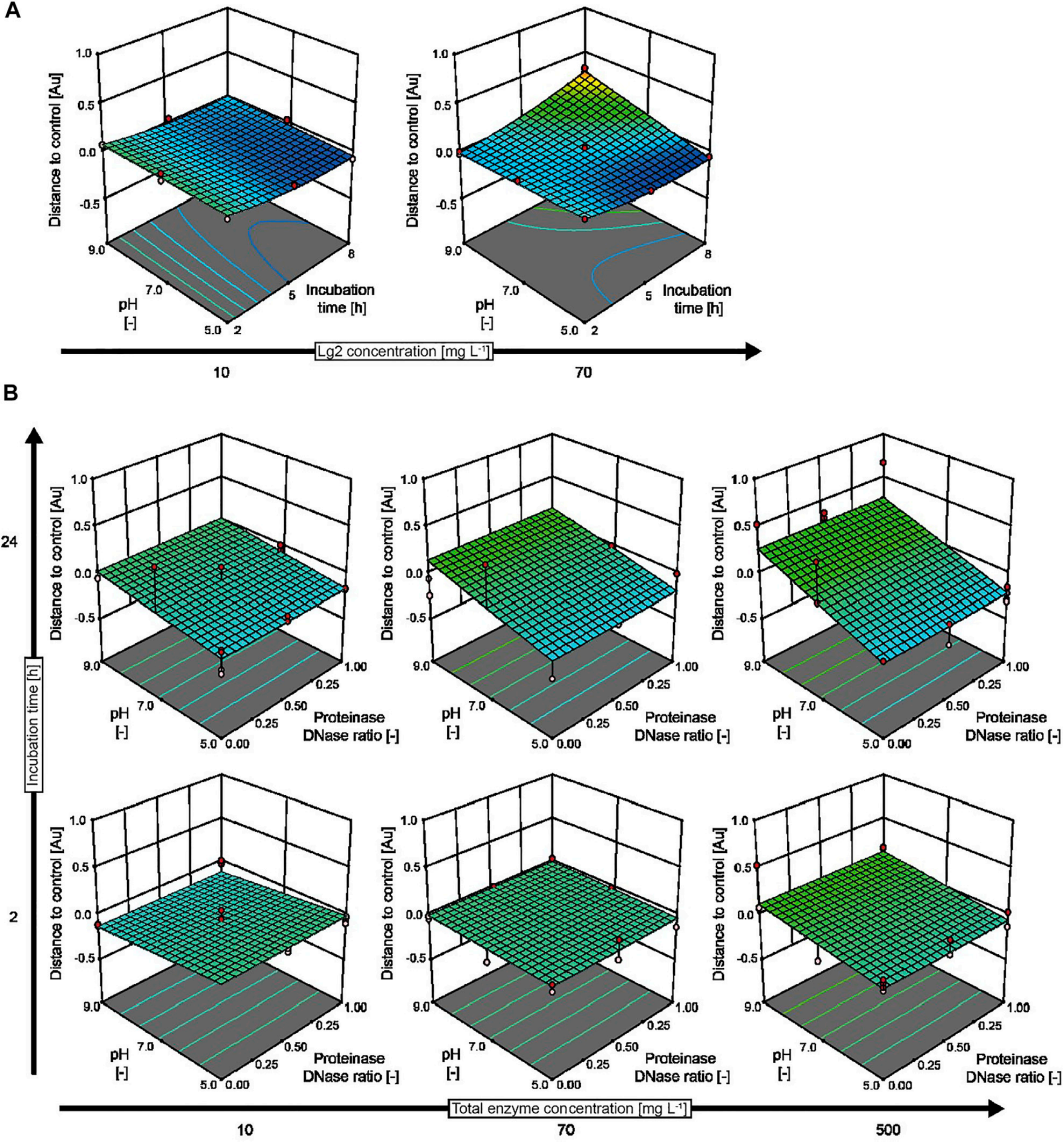
Response surface models predicting the effect of biofilm-degrading enzymes. **(A)** The effect of IMAC–purified Lg2 compared to authentic controls prepared for each of the conditions tested in an I-optimal response surface deign with 24 runs. **(B)** Combined action of proteinase K and DNase I treatment on wild-type *B. subtilis* biofilms based on a D-optimal combined mixture design with 153 runs conducted in three blocks. The experimental designs covered pH values in the range of 5.0–9.0, enzyme concentrations in the range of 10–70 mg L^−1^ (Lg2) or 10–500 mg L^−1^ (proteinase K and DNase I), and incubation periods in the range of 2–8 h (Lg2) or 2–24 h (proteinase K and DNase I). The assay buffer was 50 mM di-sodium hydrogen phosphate, and 100 mM sodium chloride (Lg2) or 50 mM TRIS (pH 7.0–9.0) or MES (pH 5.0), with 5 mM calcium chloride, 5 mM magnesium chloride (proteinase K and DNase I). Membranes with an evaporation rate of 544 g m^−2^ d^−1^ were used for all experiments.

When incubating biofilms with IMAC–purified Lg2, we observed the strongest biofilm-degrading activity at an enzyme concentration of 70 mg L^−1^, an acidic pH of 5.0, and an incubation time period of 5–8 h, resulting in the removal of ∼50% of the biofilm compared to an untreated control (Figure 3A). The low activity of Lg2 matches with the previously reported weak biofilm-degrading activity against *Staphylococcus epidermidis* (Gökçen, 2016). The acidic pH optimum agreed with the activity optimum (pH 5.0) reported for dispersin B (Kaplan and Donelli, 2014), which has 29% sequence identity to Lg2. Interestingly, biofilm formation increased at an alkaline pH (9.0) or with longer incubation time (>8 h) even in the presence of Lg2. A visible precipitate formed in the assay buffer under these conditions, probably reflecting the denaturation of Lg2 and/or remaining *N. benthamiana* host cell proteins.

We next compared the biofilm-degrading activity of Lg2 to that of the commercially available enzymes proteinase K and DNase I (Figure 3B), testing mixtures of both enzymes as previously suggested (Waryah et al., 2017) because *B. subtilis* biofilms have been shown to contain >1,000 bp DNA strands as well as proteins (Romero et al., 2018). Both enzymes were able to degrade experimental biofilms (85% removal) at a concentration of 500 mg L^−1^ when incubated for more than 24 h in an acidic buffer (pH 5.0). However, the biofilm formed by wild-type *B. subtilis* was unaffected by either enzyme at a concentration of 10 mg L^−1^ and with short incubation time (2 h). Increased biofilm formation was observed at pH 9.0 in combination with long incubation time (24 h) and high enzyme concentrations (500 mg L^−1^), as observed for Lg2. This may reflect the precipitation of proteinase K close to its isoelectric point of 8.9 (Ebeling et al., 1974) and the limited stability of DNase I outside the pH range 5–7. No synergistic effect between proteinase K and DNase I was observed during the degradation of *B. subtilis* biofilms (Supplementary Table S4). We speculate that the broad-spectrum activity of proteinase K may have inactivated DNase I during the assay. Such off-target effects could be limited in the future by selecting a more specific protease. Also, proteinase K has previously been identified as a potential biofilm-enhancing enzyme for a highly hydrophobic strain of *Rhodococcus ruber*, e.g., by inactivating enzymes involved in the regulation of the biofilm growth (Gilan and Sivan, 2013), underlining the ambivalent effect that nonspecific proteases may have on biofilm formation (Conlon et al., 2013).

Others have reduced *Staphylococcus aureus* biofilm formation using antimicrobial peptides at concentrations of 25 nM, corresponding to about 0.1 mg L^−1^, given a size of ∼40 amino acids (Segev-Zarko et al., 2015). Secondary metabolites like cytochalasins from ascomycetes reduced biofilm formation of *S. aureus* by 20–90% when applied at concentrations of 32–250 mg L^−1^ (Yuyama et al., 2018). A similar biofilm reduction of >90 was observed when ∼200 mg L^−1^ of secondary metabolites of the plant *Dodonaea viscosa* was added to *S. mutans* (Ngabaza et al., 2018). Functionalizing surfaces with biofilm-degrading enzymes such as glycoside hydrolase can reduce *Pseudomonas aeruginosa* biolayer formation by more than 99% (Asker et al., 2018). Such immobilization is mostly limited to artificial surfaces such as urinary catheters (Ivanova et al., 2015), whereas it can hardly be applied to biological surfaces *in vivo*. The screening assay we present here can help identify biofilm-degrading enzymes that can complement existing approaches using antimicrobial peptides and find interesting proteins for surface functionalization.

### Characterization of Mutant Strains of Biofilm-Forming Bacteria

**FIGURE 4:**
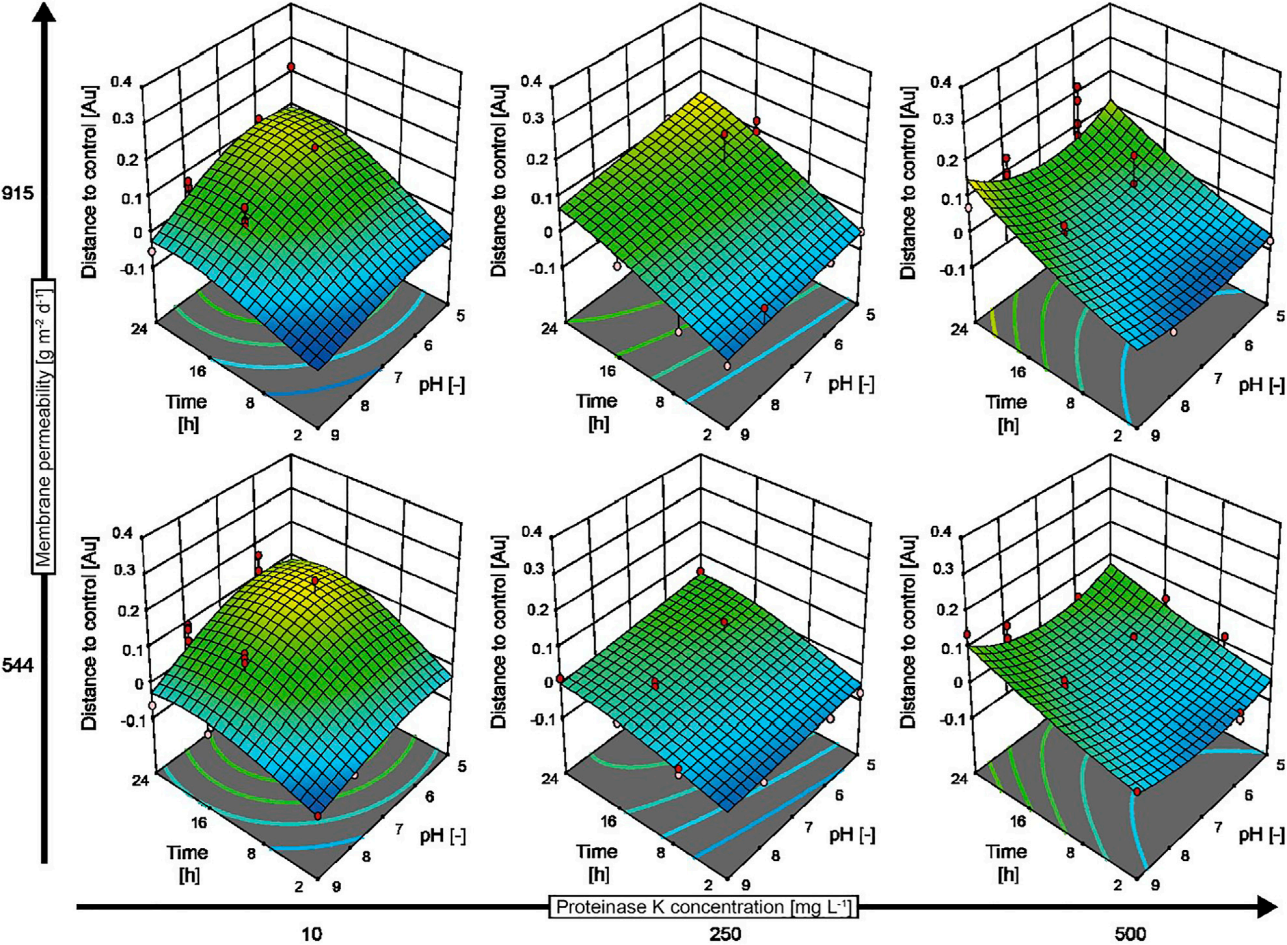
Response surface model predicting the behavior of *B. subtilis* WB800N biofilms in response to treatment with proteinase K. An I-optimal split-plot design with 128 runs was obtained in two blocks. The data were calculated by subtracting the signal measured for control biofilms (incubated in assay buffer without enzyme) from the signal measured for enzymatically treated biofilms. Negative values indicate a reduction in biofilm thickness, whereas positive values denote an increase in biofilm formation compared to the control. Experimental data are displayed as red (above the model prediction) and pink (below the model prediction) dots in the graphs. The assay buffer was 50 mM MES (pH 5.0) or 50 mM TRIS (pH 7.0–9.0) and 5 mM calcium chloride.

Mutations that cause enzyme, e.g., protease, deficiencies can change the properties of biofilms formed by microbes (O’Neill et al., 2007). For example, *B. subtilis* knockout strain WB800N lacks eight extracellular proteases (Jeong et al., 2018), reducing its biofilm-forming capability compared to wild-type *B. subtilis* (Bindel Connelly et al., 2004). However, the addition of exogenous proteases such as proteinase K can restore the biofilm-forming capability of *B. subtilis* WB800N (Bindel Connelly et al., 2004). We tested whether our automated screening assay could reproduce this behavior and can thus correctly characterize the impact of biofilm-degrading enzymes on such mutant strains by investigating the effects of different proteinase K concentrations, pH values, incubation periods, and evaporation-controlling membranes on *B. subtilis* WB800N (Figure 4). As expected, the amount of biofilms per well increased by 50–100% in the presence of low concentrations of proteinase K (10 mg L^−1^) compared to biofilms in the assay buffer without proteinase K during all but the shortest incubation periods. Only high proteinase K concentrations >250 mg L^−1^ combined with low-to-intermediate incubation periods (up to 10 h) and close to neutral pH removed up to 80% of the biofilm (Figure 4, dark blue areas). This pH optimum was in good agreement with the previously reported high activity of proteinase K at neutral-to-alkaline pH (Ebeling et al., 1974). The incomplete removal of biofilms by proteinase K was unsurprising because this enzyme can only remove the protein fraction of biofilms but not components such as PNAG and did not fully remove the biofilm formed by wild-type *B. subtilis* as stated above. Faster evaporation with a membrane of 915 g m^−2^ d^−1^ was also associated with increased variation (average standard deviation = 0.040, n = 44) compared to a membrane of 544 g m^−2^ d^−1^ (average standard deviation = 0.026, n = 44), which complicates evaluation of experimental data with mathematical models (Figure 4, comparing upper row and lower row). This is important because more evaporation is typically observed at the edges of multiwell plates, which can cause the misinterpretation of assay results if not prevented (by careful sealing) or taken into account (by introducing systematic corrections). The reactions can also be constrained to the internal wells, but this reduces the overall capacity of multiwell plates and thus the throughput of the screening assay (Wenderska et al., 2011).

## Conclusion

We have identified cultivation conditions that allow for the reproducible formation of experimental biofilms by *B. subtilis*, using the chemically defined medium MSgg without antibiotics. Using these experimental biofilms, we established an automated high-throughput assay for the screening of candidate biofilm-degrading enzymes. The assay allowed us to rapidly characterize commercially available enzymes such as proteinase K and DNase I, as well as a novel dispersin B–like model enzyme Lg2. While the typical transient expression approaches are restricted to a single compartment (Kovalskaya et al., 2016; Kovalskaya et al., 2019), we screened multiple cell compartments to utilize the full flexibility of plant-based expression systems. This unlocks the potential to increase target protein accumulation.

The purification of recombinant biofilm-degrading enzymes was achieved conveniently using a His_6_ tag for capture on IMAC resin. However, our data showed that enzyme activity can also be screened in crude extracts, making the assay simpler and more compatible with automation. Interestingly, the depth filtration step limited the overall yield of the purification process due to low product recovery. It is unclear whether these losses are a general property of biofilm-degrading enzymes, for example, because they bind to the resin or cellulose parts of the filter that may share structural features with biofilms. In any case, the maximum Lg2 accumulation level was 20 mg kg^−1^, which is in the intermediate range for transient expression and could potentially be improved by the use of silencing suppressors (Garabagi et al., 2012). It will be interesting to screen more biofilm-degrading enzymes in the future, allowing for the analysis of potential synergistic activities between enzymes with different substrate specificities, such as combinations of DNases and glycosidases (Banar et al., 2019; Cherny and Sauer, 2019).

## Data Availability

The raw data supporting the conclusions of this article will be made available by the authors, without undue reservation.

## Abbreviations

BY-2, Bright Yellow 2; EPS, exopolysaccharides; Lg2, Lysobacter gummosus glyco 2; PCPs, plant cell packs; PNAG, poly-N-acetyl-β-(1,6)-glucosamine; UTR, untranslated region.
