# Supplementary material for: Expression of Biofilm-Degrading Enzymes in Plants and Automated High-Throughput Activity Screening Using Experimental Bacillus subtilis Biofilms
Source: Front Bioeng Biotechnol. 2021 Sep 21;9:708150. doi: 10.3389/fbioe.2021.708150 (PMC8490750; doi:10.3389/fbioe.2021.708150)
Supplement: Supplementary file 1 [file Presentation1.pdf]

Supplementary materials

Table S1: Overview of cloned Lg2 expression vectors that were screened in BY-2 PCPs and differentiated *N. benthamiana* plants.

Construct ID	5′UTR	Signal peptide	C-terminal tag /signal sequence	Target compartment
000198	CHS	None	Hexa-his	Cytosol
000199	CHS	LPH	Hexa-his	Apoplast
000200	CHS	TP	Hexa-his	Plastids
000201	CHS	LPH	Hexa-his, KDEL	ER
000202	omega	None	Hexa-his	Cytosol
000203	omega	LPH	Hexa-his	Apoplast
000204	omega	TP	Hexa-his	Plastids
000205	omega	LPH	Hexa-his, KDEL	ER
000206	TL	None	Hexa-his	Cytosol
000207	TL	LPH	Hexa-his	Apoplast
000208	TL	TP	Hexa-his	Plastids
000209	TL	LPH	Hexa-his, KDEL	ER

CHS – *Petroselinum hortense* chalcone synthase gene 5′UTR; ER – endoplasmic reticulum; KDEL – SEKDEL sequence for target protein retention in the ER; LPH – leader peptide sequence of the murine monoclonal antibody mAb24 heavy chain; omega – omega prime sequence from *Tobacco mosaic virus*; TL – *Tobacco etch virus* leader sequence.

Table S2: Comparison of the biofilm variance measured after incubation in 50 mM MES (pH 5.0) or TRIS (pH 7.0-9.0) containing 5 mM calcium chloride at different pH values and incubation times using the *F*-test (OriginPro 2015). Conditions resulting in a significantly different variance are highlighted in bold font.

Condition 1	$n_{Condition\ 1}$	Condition 2	$n_{Condition\ 2}$	alpha	p-value
pH 7.0, 2.0 hours	3	pH 5.0, 2.0 hours	3	0.05	0.09281
pH 7.0, 9.5 hours	3	pH 5.0, 9.5 hours	3	0.05	0.81053
pH 7.0, 17.0 hours	3	pH 5.0, 17.0 hours	3	0.05	0.92671
**pH 7.0, 24.0 hours**	**3**	**pH 5.0, 24.0 hours**	**3**	**0.05**	**0.01632**
pH 7.0, 2.0 hours	3	pH 9.0, 2.0 hours	3	0.05	0.50922
pH 7.0, 9.5 hours	3	pH 9.0, 9.5 hours	3	0.05	0.68004
pH 7.0, 17.0 hours	3	pH 9.0, 17.0 hours	3	0.05	0.36234
**pH 7.0, 24.0 hours**	**3**	**pH 9.0, 24.0 hours**	**3**	**0.05**	**0.00578**
pH 7.0, 24.0 hours	3	pH 7.0, 2.0 hours	3	0.05	0.45459
pH 7.0, 24.0 hours	3	pH 7.0, 9.5 hours	3	0.05	0.29505
pH 7.0, 24.0 hours	3	pH 7.0, 17.0 hours	3	0.05	0.46066
pH 5.0, 24.0 hours	3	pH 5.0, 2.0 hours	3	0.05	0.09476
pH 5.0, 24.0 hours	3	pH 5.0, 9.5 hours	3	0.05	0.06277
pH 5.0, 24.0 hours	3	pH 5.0, 17.0 hours	3	0.05	0.06173
**pH 9.0, 24.0 hours**	**3**	**pH 9.0, 2.0 hours**	**3**	**0.05**	**0.00498**
**pH 9.0, 24.0 hours**	**3**	**pH 9.0, 9.5 hours**	**3**	**0.05**	**0.01710**
pH 9.0, 24.0 hours	3	pH 9.0, 17.0 hours	3	0.05	0.08382

Table S3: Impact of different assay buffer compositions on experimental biofilms over time. Linear fits were calculated for different assay buffers and pH values displayed in Figure S2 using OriginPro 2015. The slope and standard error of the respective linear fit were derived as a measure for the impact of the assay buffer on the biofilm.

Buffer [-]	pH [-]	Slope [-]	Standard error [-]
50 mM TRIS or MES,	5.0	0.00509	0.00162
100 mM sodium	7.0	0.00414	0.00276
chloride	9.0	0.03914	0.01432
50 mM TRIS or MES,	5.0	0.00559	0.00283
5 mM calcium	7.0	0.00100	0.00209
chloride	9.0	0.02261	0.01029
50 mM disodium	5.0	0.00321	0.00031
hydrogen phosphate,	7.0	-0.00171	0.00013
100 mM sodium	9.0	0.00363	0.00026
chloride			

Table S4: Model factors with a significant influence on experimental biofilms formed by wild-type *B. subtilis* during incubation with Lg2 or a combined treatment with proteinase K and DNase I (linear mixture) as identified by analysis of variance.

**Lg2**			**Proteinase K and DNase I**		
**Source**	**F-value**	**p-value**	**Source**	**F-value**	**p-value**
Model	15.52	< 0.0001	Model	7.73	< 0.0001
A (Lg2 concentration)	4.60	0.0488	Linear mixture	0.6677	0.4153
B (pH)	17.82	0.0007	C (pH)	13.40	0.0004
C (incubation time)	0.0102	0.9209	E (enzyme concentration)	7.83	0.0059
AB	8.10	0.0123	AC	0.1222	0.7272
AC	18.90	0.0006	AD	0.2949	0.5880
BC	14.42	0.0018	AE	2.05	0.1542
C^2	7.62	0.0146	CD	8.80	0.0036
ABC	9.16	0.0085	CE	12.50	0.0006
---	---	---	C^2	6.33	0.0130
---	---	---	ACD	0.6499	0.4216
---	---	---	ACE	1.58	0.2113
---	---	---	ADE	0.0073	0.9322
---	---	---	AC^2	6.98	0.0092
---	---	---	ACDE	6.11	0.0146
Coefficients of determination	R^2	0.8922	Coefficients of determination	R^2	0.4451
	Adjusted R^2	0.8347		Adjusted R^2	0.3875
	Predicted R^2	0.6590		Predicted R^2	0.2765

Factors with a non-significant influence on the responses ($p > 0.05$) were removed from the model unless they were required to maintain model hierarchy. With a difference <0.2, the values of R^2, adjusted R^2 and predicted R^2 for the Lg2 model were in reasonable agreement with each other. The comparatively low values of R^2, adjusted R^2 and predicted R^2 in the model for a combined proteinase K and DNase I treatment were attributed to high standard deviations at an incubation pH of 9.0, due to the limited stability of proteinase K (pI = 8.9) and DNase I (precipitation at pH 9.0) under these conditions.

Table S5: Model factors with a significant influence on experimental biofilms formed by *B. subtilis* WB800N during treatment with proteinase K as identified by restricted maximum likelihood analysis.

**Source**	**F-value**	**p-value**
Whole-plot	8.89	0.0035
A (Membrane permeability)	8.89	0.0035
Subplot	25.06	< 0.0001
B (pH)	23.30	< 0.0001
C (incubation time)	170.75	< 0.0001
D (proteinase K concentration)	43.70	< 0.0001
AC	8.02	0.0055
AD	2.73	0.1015
BD	31.55	< 0.0001
CD	0.0175	0.8951
B^2	0.0000	0.9950
C^2	20.37	< 0.0001
D^2	0.0876	0.7678
AD^2	10.61	0.0015
B^2D	16.39	< 0.0001
C^2D	12.13	0.0007
Coefficients of determination	R^2	0.8070
	Adjusted R^2	0.7771
	Predicted R^2	n.a.

Factors with a non-significant influence on the responses ($p > 0.05$) were removed from the model unless they were required to maintain model hierarchy. A predicted R^2 is not available for the model because a split-plot design was used, for which this metric cannot be calculated. With a difference <0.2, the values of R^2, adjusted R^2 and predicted R^2 were in reasonable agreement with each other.

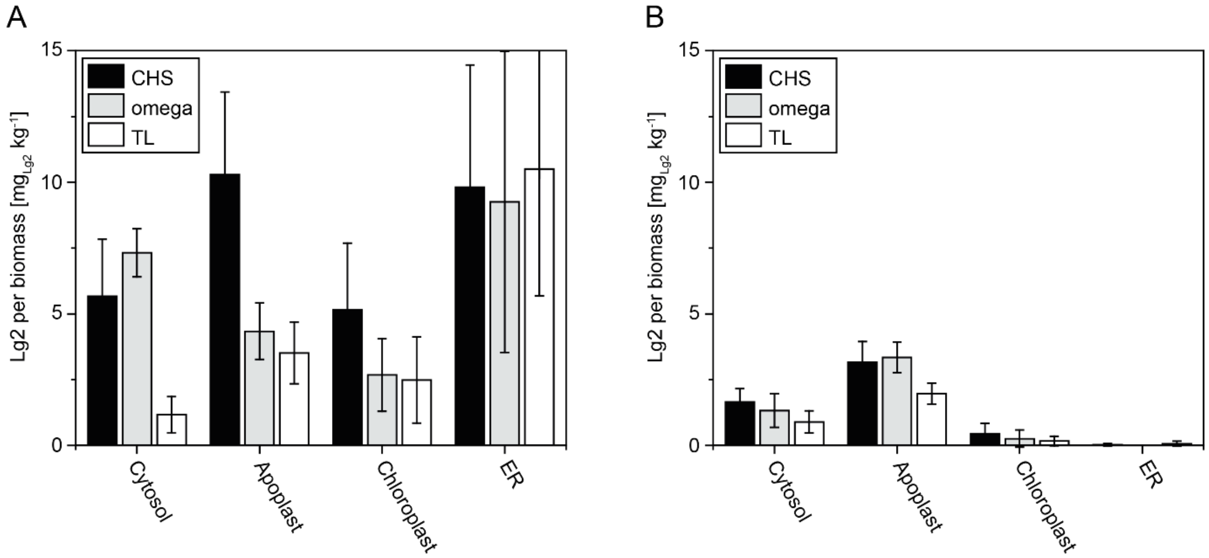

Figure S1: Comparison of Lg2 accumulation levels in differentiated plants and PCPs. A. Lg2 accumulation per biomass in differentiated *N. benthamiana* plants at 5 dpi based on densiometric evaluation of dot blots using a His6-specific primary antibody and an AP-labeled goat anti-rabbit secondary antibody. Data are means ± standard deviation for n = 3 (or n = 6 for ER constructs) individual plants. B. Lg2 accumulation per biomass in PCPs at 3 dpi based on densiometric evaluation of dot blots using the same antibodies as in A. Data are means ± standard deviation for n = 4 individual PCPs.

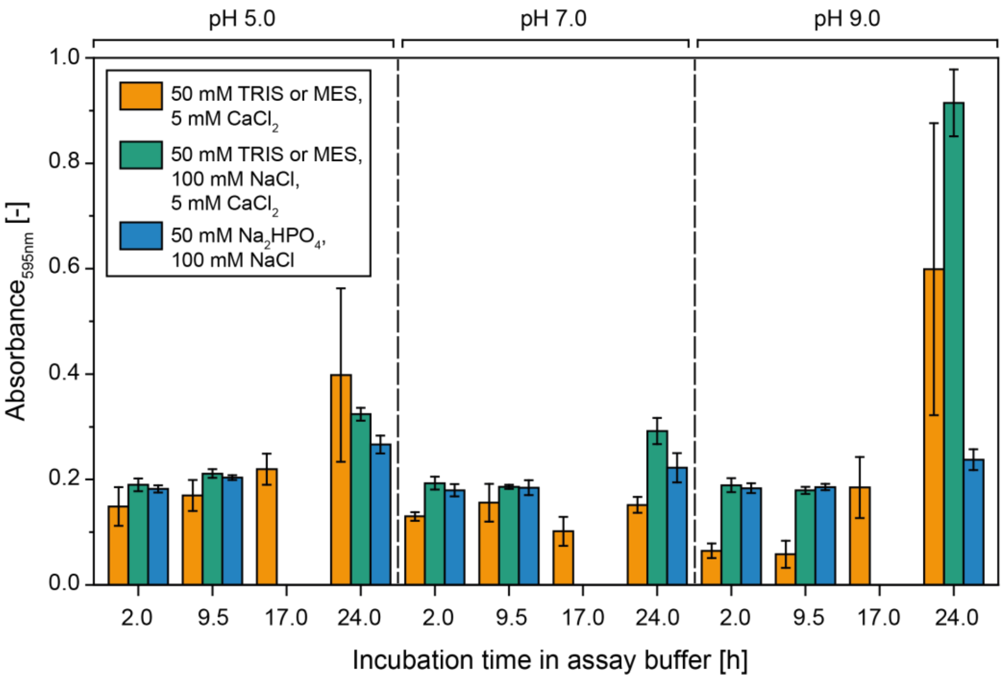

Figure S2: Wild-type *B. subtilis* biofilms pre-formed for 24 h in MSgg medium were incubated in buffers frequently used in the context of biofilm degradation. Data for an incubation time of 17 h are unavailable for all buffers due to an error in the robotic device. Data are means ± standard deviations (n = 3-7).

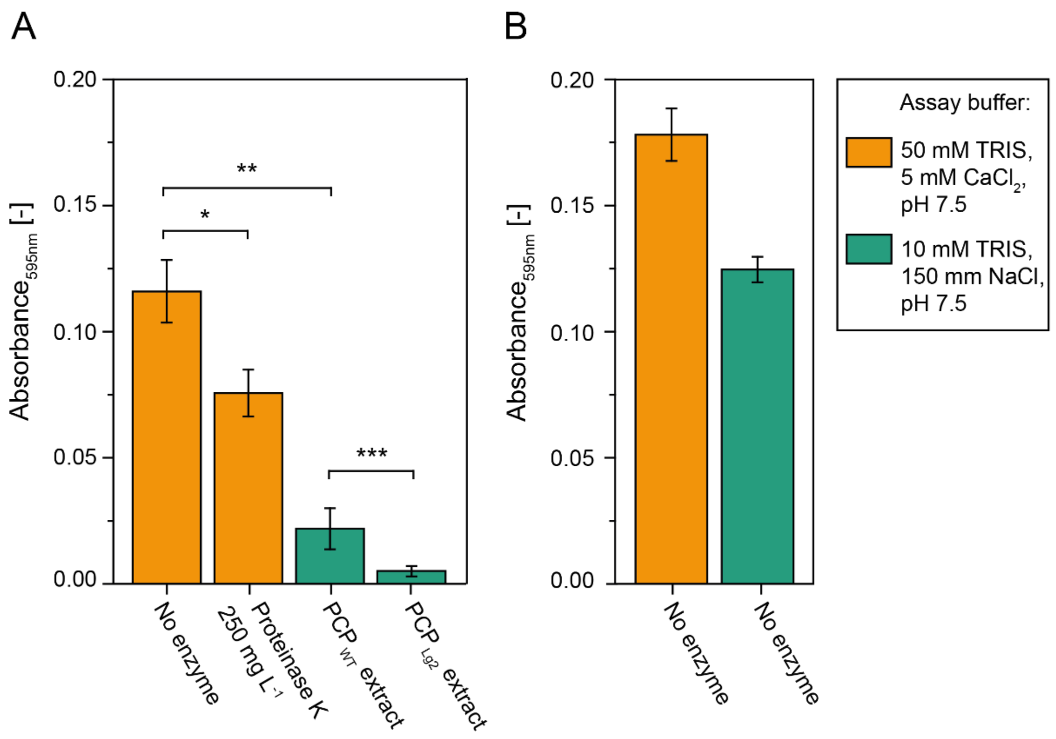

Figure S3: Effect of test conditions on biofilm staining. A. Staining intensity of untreated biofilms and those exposed to proteinase K, extracts from wild-type (WT) PCPs, or crude Lg2. B. Biofilms pre-formed for 24 h in MSgg medium were exposed to assay buffers for 10 h at 37°C using a membrane with an evaporation rate of 544 g m^{-2} d^{-1}. Data are means ± standard deviations for 4–8 biological replicates (A) or 16 replicates (B). Statistical significance: *p <0.001 (two-sided two-sample t-test, α = 0.05, n= 4 or 8); ** p <0.001 (two-sided two-sample t-test, α = 0.05, n=4 or 8); ***p = 0.023 (two-sided two-sample t-test, α = 0.05, n=4, significantly different variance).
